# Genetic Variants Involved in Metformin Pharmacokinetics in the Chilean Population

**DOI:** 10.3390/genes17091114

**Published:** 2026-09-14

**Authors:** José P. Miranda, Gigliola Alberti, Ana Pereira, Juan Cristóbal Gana, José L. Santos

**Affiliations:** 1 Department of Nutrition, Diabetes and Metabolism, School of Medicine, Pontificia Universidad Católica de Chile, Santiago 8331150, Chile; jose.miranda@uc.cl; 2 Dirección de Desarrollo Médico y Académico, Bupa Chile, Santiago 7550000, Chile; 3 Department of Pediatric Gastroenterology and Nutrition, Division of Pediatrics, School of Medicine, Pontificia Universidad Católica de Chile, Santiago 8331150, Chile; g.alberti@uc.cl (G.A.); jcgana@gmail.com (J.C.G.); 4 Institute of Nutrition and Food Technology (INTA), University of Chile, Santiago 7830490, Chile; apereira@inta.uchile.cl; 5 Department of Health Sciences, Institute for Sustainability and Food Chain Innovation (IS-FOOD), Public University of Navarre, 31008 Pamplona, Spain; 6 Navarra Institute for Health Research (IdiSNA), 31008 Pamplona, Spain; 7 Doctoral Program in Epidemiology, School of Medicine, Pontificia Universidad Católica de Chile, Santiago 8331150, Chile

**Keywords:** metformin, diabetes, pharmacogenetics, genetic variants, pharmacokinetics

## Abstract

Objective: To estimate the frequency and potential impact of genetic variants involved in metformin pharmacokinetics, including gastrointestinal absorption, renal elimination, and hepatic transport in the Chilean population. Subjects and methods: The frequency of metformin-related gene variants was estimated using data from a genome-wide, exome-focused genotyping array in 918 participants of the Chilean Growth and Obesity Cohort Study (GOCS). Allele frequencies in Chileans were compared with those published worldwide, and the degree of Amerindian/European ancestry was assessed. A predictive bioinformatic analysis was performed on relevant genetic variants using PolyPhen-2. Results and Discussion: The genetic polymorphism (p.Leu125Phe; p.L125F; rs77474263; C>T) in the SLC47A1 gene (MATE1 transporter) showed a much higher frequency of the 125Phe (T-allele) in the Chilean population (15.3%) than in non-American populations, being virtually absent in Africa, Europe, or Asia. The 125Phe variant was associated with a higher percentage of Amerindian ancestry in the Chilean population. This result is consistent with a previous report indicating that this variant is enriched in Native American populations, given its high frequency among Mestizo and Indigenous populations in Mexico. The functional variant p.125Phe has been previously suggested to promote plasma metformin accumulation, mitochondrial dysfunction, and increased plasma lactate concentrations by altering renal metformin elimination. Conclusions: The missense variant p.Leu125Phe (rs77474263) in SLC47A1 is frequent in Chile and Mexico, and apparently absent in non-American populations. In homozygosis, the p.125Phe variant has been previously associated with impaired renal drug elimination, metformin systemic accumulation, and hyperlactatemia.

## 1. Introduction

Metformin is the first-line oral drug recommended for the treatment of hyperglycemia in Type 2 Diabetes Mellitus (DM2) [1,2,3]. According to the 2016–2017 National Health Survey, the prevalence of metformin use among individuals older than 17 years in Chile was 9.8%, making it the fourth most commonly used medication in the country [4]. This high number of metformin users is a direct consequence of the epidemic dimensions of obesity and DM2 in Chile and around the world. Metformin acts without altering insulin secretion, thereby minimizing the risk of hypoglycemia [5], and is a safe and inexpensive drug. Besides the widely reported effects of metformin in DM2, it has been proposed that this drug has additional pleiotropic benefits as an anti-tumorigenic agent and confers protection against cardiovascular disease, aging, and neurocognitive decline [6,7]. Metformin also deserves special mention for its off-label use in managing obesity, regulating appetite, and treating metabolic dysfunction-associated steatotic liver disease (MASLD) [8,9,10,11,12,13].

Despite its use for decades in medicine, the mechanism of metformin’s hypoglycemic action has not been fully elucidated [14,15,16], with several suggested hypotheses: (a) metformin has been reported to inhibit hepatic gluconeogenesis by phosphorylation and activation of AMPK [17]; (b) metformin may inhibit the mitochondrial enzyme glycerophosphate dehydrogenase, which may alter the hepatocellular redox state, which would, in turn, lead to a reduction in lactate and glycerol to hepatic gluconeogenesis [18]; (c) a metformin may inhibit the mitochondrial electron transport chain at complex I and IV [16]; (d) metformin may play an important role in the gut, given that its antihyperglycemic effect is more significant when administered orally instead of intravenously, possibly via distal small intestine-mediated mechanisms [19,20]; (e) metformin reduces appetite by enhancing growth differentiating factor 15 (GDF15) [8,9]; (f) metformin may act as an anti-aging drug through the previously mentioned mechanisms increasing insulin sensitivity and decreasing mTOR signaling, oxidative stress, genetic instability and inflammation [6,21], and (g) metformin has been described to alter the intestinal microbiota composition, mainly by promoting an increase in the population of butyrate, propionate, succinate, and mucin-producing bacteria, with a positive hypoglycemic and anti-inflammation effects [22]. Several Genome-wide association studies (GWAS) and candidate-gene studies have identified gene variants associated with response to metformin treatment in patients with Type 2 diabetes, with significant signals of association for GLUT2, TCF7L2, ATM, PRPF31, CPA6, STAT3, ENOSF1, and OSMR [23,24,25,26,27,28,29].

**Figure 1:**
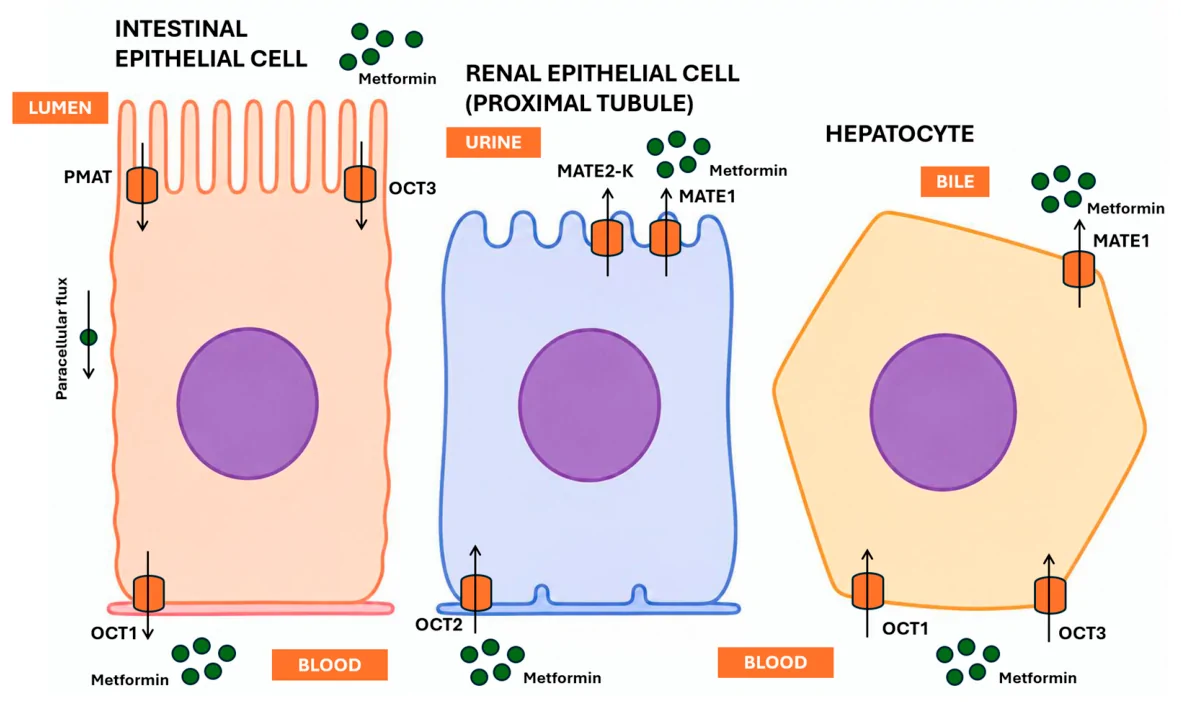
Transporters involved in metformin pharmacokinetics. Metformin is a hydrophilic molecule; as such, it uses transporters to cross cell membranes. It is absorbed by enterocytes through transporters located in the apical membrane, PMAT (plasma membrane monoamine transporter) and OCT3 (organic cation transporter 3), encoded by genes SLC29A4 and SLC22A3, respectively. Once inside the enterocyte, metformin uses transporter OCT1 (SLC22A1; located in the basolateral membrane) to enter the bloodstream. In humans, a minor fraction of metformin may enter hepatocytes via the OCT1 and OCT3 transporters, with small amounts excreted into bile via the canalicular MATE1 transporter. Metformin is incorporated into renal epithelial cells via OCT2 (SLC22A2; basolateral membrane) and is eliminated via MATE1/MATE2-K (apical membrane). Of these, MATE1 is more important than MATE2-K (SLC47A2) due to MATE1’s higher expression and higher metformin affinity. Because metformin clearance exceeds the glomerular filtration rate, active tubular secretion mediated by the coordinated action of OCT2 and MATE1/MATE2-K represents the dominant mechanism of its renal elimination. Additionally, MATE1 is also expressed at low levels in enterocytes, playing a minor role in metformin efflux back into the intestinal lumen [31]. Allele frequencies of variants in genes involved in metformin pharmacokinetics may differ significantly among populations, depending on ethnicity, admixture profile, and migration history [31]. This study aims to estimate the frequency of genetic variants associated with metformin pharmacokinetics in the Chilean population.

In addition to metformin’s efficacy in maintaining glucose homeostasis in DM2 patients, another approach to studying the drug’s effects is to evaluate its pharmacokinetics [1,30]. After ingestion, metformin reaches its peak plasma concentration at 4–8 h, with 50–60% bioavailability and 90% renal excretion in humans. Several allelic variants have been reported in genes involved in metformin pharmacokinetics [31], including SLC22A1 (encoding OCT1), SLC22A2 (OCT2), SLC22A3 (OCT3), SLC29A4 (PMAT1), SLC47A1 (MATE1), and SLC47A2 (MATE2) (Table 1 and Figure 1). The main adverse effects of metformin are linked to its transport in the gut (nausea, abdominal discomfort, and osmotic diarrhea) and renal clearance mechanisms, leading to systemic drug accumulation. A relatively high percentage of patients treated with metformin (around 30%) show gastrointestinal intolerance (GI), which can lead some patients to stop their medication [5]. In humans, a minor fraction of metformin enters hepatocytes via the OCT1/OCT3 transporters, with small amounts (typically <1%) excreted into bile via the canalicular MATE1 transporter. Metformin is not modified by the liver, does not bind to plasma proteins, and is primarily eliminated by the kidneys via glomerular filtration and active tubular secretion. Circulating metformin may increase due to impaired renal clearance. In this situation, high intracellular drug concentration may enhance inhibition of mitochondrial respiratory complexes I and IV [16], increasing cytosolic NADH, decreasing ATP synthesis, and shifting cellular metabolism from pyruvate to lactate, thereby increasing the risk of metformin-associated lactic acidosis (MALA) [32]. This rare event is characterized by high anion-gap metabolic acidosis, elevated lactate (>5 mmol/L), and arterial pH < 7.35 [32]. While events of MALA are rare, occurring in ~3–10 cases per 100,000 patient-years, the condition is life-threatening and associated with an estimated ~35% mortality rate. In this line, phenformin hydrochloride, another biguanide in the same class as metformin, was withdrawn from clinical use because of its strong association with lactic acidosis (40–64 cases per 100,000 patient-years) [32].

## 2. Subjects and Methods

### 2.1. The Chilean Growth and Obesity Cohort Study (GOCS)

A cross-sectional study within the Chilean “Growth and Obesity Cohort Study” (GOCS) was conducted to estimate allele/genotype frequencies of genetic variants associated with metformin action. GOCS is a Chilean cohort initiated in 2006 among children aged 2.6–4 years attending kindergartens in Chile’s Preschool Program (JUNJI and JUNAEB) in six counties of the city of Santiago (Chile), representing low- to middle-income households in the city. The inclusion criteria were single-birth children with birth weights between 2500 and 4500 g and without physical or psychological alterations that could affect growth. The sample comprised 1195 participants, of whom 918 were included in the genotyping analysis [33]. In this cohort, multiple growth and maturational development-related phenotypes have been evaluated, as well as nutrition-related disease risk factors. Written informed consent from parents or guardians and children’s assent were obtained for GOCS participants. The study was approved by the Ethics Committee of the School of Medicine of the Pontificia Universidad Católica de Chile, and INTA-University of Chile.

### 2.2. Genotyping with MEGA-Illumina Array

Genotyping was performed in 918 children of the GOCS cohort using the genome-wide exome-focused Infinium Multi-Ethnic Global BeadChip DNA microarray (Illumina, San Diego, CA, USA) (MEGA-Illumina). This microarray enables the determination of genotypes for 1,779,819 genetic variants, primarily single-nucleotide variants (SNVs). For quality-control analysis, the GenomeStudio v2.0.3 (Illumina) and PLINK v1.9 programs were used. The participant exclusion criteria were based on genotyping quality control (SNVs with a call rate < 0.99 were excluded), high kinship relationships, or extreme deviations from Hardy–Weinberg equilibrium. Figure S1 shows the flow analysis in the GOCS participants and genotyping quality control (QC).

### 2.3. Ethnic Ancestry Estimation

The percentage of native American (Mapuche) ethnic ancestry of GOCS participants was estimated from genome-wide genotyping using genetic markers on the MEGA-Illumina microarray and principal component analysis [33,34]. For this calculation, the highly unbalanced linkage genetic regions were initially removed (cut-off value of r^2^ = 0.2), with a window of 50 Kb and a step of 5 SNV, which gave as many as 233,649 variants that were finally analyzed with the ADMIXTURE 1.4.0 software to determine global ancestry [33,34] with *K* = 5 populations. The global Native American component of the Chilean GOCS was previously estimated at 61.4%, primarily Mapuche (55.3%), with a low contribution from Andean (3.84%) and Central American origin (2.2%). The global European ancestry accounted for 36%, mainly from southern Europe. There were very small contributions (<1%) from African or Asian populations [33,34]. Details on the assessment of global ancestry among GOCS participants are shown in the Supplementary Material. Table S1 shows the average ancestry proportions among GOCS participants.

### 2.4. Statistical Analysis

Allele and genotype frequencies were calculated for each variant and tested for agreement with the Hardy–Weinberg equilibrium. Association of p.Leu125Phe genotypes with the percentage of Native American (Mapuche) ancestry was assessed by a non-parametric (Kruskal–Wallis) test and by linear regression analysis using genotype and allele-dosing (additive) models, with calculation of beta coefficients and 95% confidence intervals (95%CI). Statistical analyses were carried out with the STATA 17 package (https://www.stata.com; accessed on 1 September 2026). The α = 0.05 threshold was used to declare statistical significance.

### 2.5. Selection of Genetic Variants Related to Metformin Pharmacokinetics

A search was conducted to identify genes and genetic variants relevant to metformin action, including intestinal absorption, liver transport, and renal elimination (https://www.pharmgkb.org/; accessed on 1 September 2026) (Figure 1 and Table 1). In this search, allelic variants of genes in Table 1 were included if they simultaneously fulfilled three strict conditions: (a) exonic gene variants with non-zero frequency in the Chilean population (as estimated in the Chilean GOCS), (b) missense or nonsense variants that were directly measured in the MEGA-Illumina array (not imputed variants), and (c) gene variants with very high PolyPhen scores (>0.95) suggesting functional effects on protein function.

### 2.6. Information on Allele Frequencies Across Populations

The frequencies of genetic variants across different populations worldwide were obtained from the Human Genome Diversity Project (HGDP), as reported in the Genome Aggregation Database (gnomAD v4.1.1; https://gnomad.broadinstitute.org/; accessed on 1 September 2026) and from a pharmacogenetic study of the p.L125F (MATE1) polymorphism conducted in Mexico [35].

### 2.7. Predictive Bioinformatic Analysis of Exonic Genetic Variants

The PolyPhen-2 (Polymorphism Phenotyping v2) program was used to assess the functional impact of SNVs that cause amino acid changes in protein function (https://genetics.bwh.harvard.edu/pph2/bgi.shtml; accessed on 1 September 2026).

## 3. Results

Table 2 shows allele frequencies and bioinformatics PolyPhen-2 predictions of gene variants fulfilling the strict inclusion criteria mentioned above: three SNVs in the OCT1 (SLC22A1) gene and one SNV in the MATE1 (SLC47A1). SLC22A1 variants showed the expected allele frequencies in Chile when compared to other worldwide populations, given the European-Amerindian admixture of the Chilean population. The 125Phe (T-allele) variant of the gene polymorphism rs77474263 (C>T; p.Leu125Phe) of the SLC47A1 gene was found to be virtually absent in populations outside the Americas (Europe, Africa, Asia), as reported by the Human Genome Diversity Project (HGDP) (Table 2) [35]. Allele frequency for Oceania is also zero, although only based on 28 alleles. In contrast, HGDP reported that the frequency of this variant was 11.5 in admixed Americans and 13.0 in Native Americans (accessed 26 August 2026).

**Figure 2:**
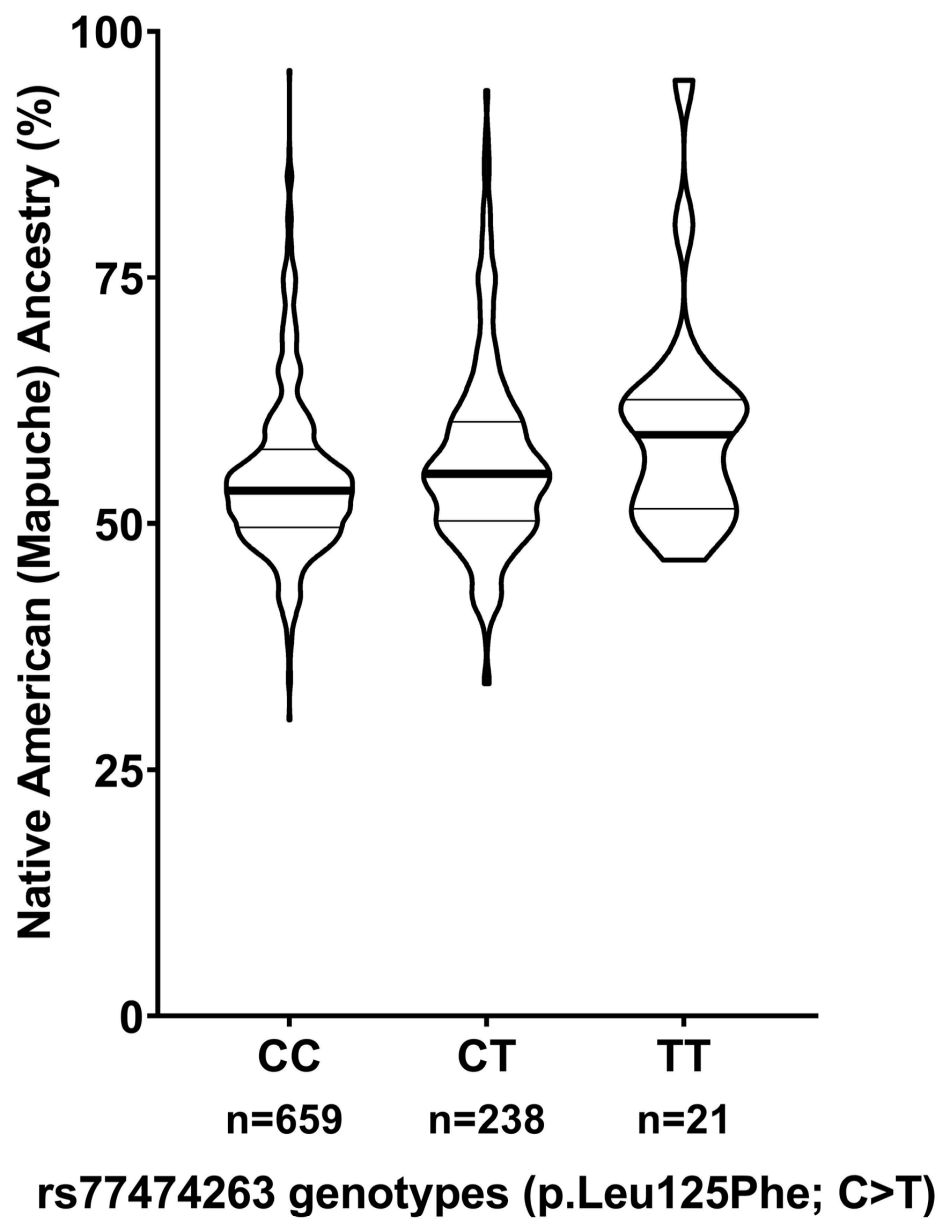
Native American (Mapuche) ancestry and SLC47A1 (MATE1) rs77474263 genotypes (p.Leu125Phe; C>T) in Chileans. The graph shows significantly higher estimates of American ancestry (Mapuche) in TT (T: minor allele), CT, or CC genotypes in the Chilean GOCS (*p* = 0.002 in the Kruskal–Wallis test and linear regression). Horizontal lines represent quartiles. Genotype frequencies were concordant with Hardy–Weinberg expectations (*p* = 0.93).

In our Chilean cohort, we found that the minor allele T of rs77474263 (C>T; p.Leu125Phe) is present at a frequency of 15.3% (2.3% of TT homozygotes), with genotype frequencies in agreement with Hardy–Weinberg expectations (*p* = 0.93; Figure 2). The median Native American ancestry (Mapuche) of the CC genotype in Chile was 53.4% (binomial 95%CI: 52.9–53.9); for the CT genotype, it was 55.1% (binomial 95%CI: 54.1–55.9), and for the homozygous TT genotype, it was 59.1% (binomial 95%CI: 52.6–62.1) (Figure 2). There were significant differences in the median Native American ancestry (Mapuche) across genotypes of this polymorphism (*p* = 0.002; Kruskal–Wallis test). Using dummy variables for each genotype and taking the CC as the reference in linear regression analysis, CT genotypes increased the mean Amerindian (Mapuche) ancestry by 1.73 percentage units (95%CI: 0.43–3.0), while TT increased by 5.1 percentage units (95%CI: 1.3–8.9) (global test for association: *p* = 0.002). Under an additive genetic model (0, 1, or 2 copies of the T allele), each additional T allele was associated with an estimated 2.0 percentage point increase in Amerindian (Mapuche) ancestry (95% CI: 0.86–3.1).

## 4. Discussion

We aimed to estimate the frequency and potential impact of functional genetic variants involved in metformin pharmacokinetics in a population-based study in Chile, and to contrast these allele frequencies with those reported worldwide (see genes in Table 1 and Figure 1). Following strict selection criteria limited to missense variants with functional effects, we ultimately focused on three polymorphisms in the OCT1 gene and one in the MATE1 gene. Our main finding reveals a relatively high frequency (15.3%) of the functional SLC47A1 (MATE1) p.Leu125Phe variant (rs77474263; C>T) in Chile, an allele virtually undetected in African, European, and Asian populations. Consistent with the high frequency of the 125Phe variant in the Mestizo Mexican population (n = 968; 16%) and in Indigenous Mexicans (n = 452; 29%) [35], we found that this variant was positively associated with the percentage of Amerindian ancestry in Chile. Based on previous research, 125Phe has been shown to be a decreased-function variant affecting protein function in studies *in vitro* and *in vivo*, and is a potential risk factor for MALA, as described in the following paragraphs [35,36,37]. This functional variant is particularly important in pharmacogenomics in Latin America, given its relatively high frequency in Mexico and Chile, and possibly in other countries in the region.

Metformin-related gastrointestinal intolerance frequently leads to treatment discontinuation [5]. The most frequent symptoms reported by patients include diarrhea and nausea, followed by flatulence, indigestion, vomiting, and abdominal distension [5]. The mechanisms involved are multifactorial and may include impaired basolateral efflux via OCT1 (SLC22A1), elevated serotonin via inhibition of the serotonin transporter (SERT), and dysbiosis [38,39]. OCT1 inhibitors (e.g., verapamil), gastrointestinal comorbidities, and decreased-function variants (R61C, M420del, G401S, G465R) double the risk of adverse effects by promoting local drug retention [40]. In our Chilean cohort and in other populations worldwide, these deleterious OCT1 alleles are present at very low frequencies (in Chile: R61C: 3.2%; G401S: 1.1%; G465R: 0.8%).

Metformin is eliminated by the kidneys through a combination of filtration and active tubular secretion via OCT2 (expressed in the basolateral membrane of renal epithelium) and MATE1/MATE2-K (expressed in the apical membrane) [30,31,41]. Metformin clearance is higher than the glomerular filtration rate, indicating that tubular secretion via MATE1 (SLC47A1) and, to a lesser extent, via MATE2-K (SLC47A2), is the dominant mechanism of metformin renal excretion [30]. Impaired MATE1 function might, on the one hand, enhance metformin efficacy by prolonging its systemic half-life, but, on the other hand, increase the risk of metformin concentration-dependent adverse effects. Plasma metformin accumulation due to non-functional MATE1 may exacerbate inhibition of mitochondrial respiratory chain, and promote increased lactate production, potentially leading to MALA [32]. In this sense, MATE1 inhibitors (e.g., cimetidine) and mutations in mitochondrial DNA (mt.3243A>G) have been proposed to trigger MALA [42,43,44]. The SLC47A1 125Phe allele (rs77474263) is a decreased-function variant (rs77474263) that impairs MATE1 transport function, as demonstrated in functional *in vitro* studies with transfected HEK-293 cells [36], and supported by *in silico* molecular dynamics analysis [37]. Notably, the same functional characterization revealed that the p.Leu125Phe variant significantly impairs MATE1-mediated transport of prototypical organic cations—including paraquat and tetraethylammonium (TEA)—while largely sparing oxaliplatin uptake. This differential transport profile was attributed to partial intracellular retention/mislocalization rather than a complete loss of function [36]. According to the Clinical Pharmacogenetics Implementation Consortium (CPIC) classification (https://www.clinpgx.org/cpic; accessed on 1 September 2026), such substrate-dependent selectivity supports the notion that 125Phe functions as a hypomorphic (decreased-function) rather than a null allele. It is also important to highlight that its clinical and toxicological relevance may reach beyond metformin to other xenobiotics cleared through renal MATE1 pathways. Interestingly, the Mate1 knockout (-/-) mouse exhibits marked hyperlactatemia and elevated plasma metformin levels following metformin administration [37]. It was reported that after 2 h of metformin administration, there was a much sharper increase in lactatemia in a pediatric patient homozygous for rs77474263 TT (approximately a 3.5-fold increase) compared with no increase in C-allele carriers [35]. This seminal observation in humans, although based on only one TT patient, is fully consistent with the effects of metformin in the Mate1 knockout (-/-) mouse [37], with no effects in TC heterozygotes [35,45].

It has been reported that the MATE1 p.Leu125Phe (L125F; C>T) substitution is a relatively frequent variant in the Mexican Mestizo population and even more so in indigenous Mexican populations [35]. The rs77474263 125Phe variant (T-allele) was also relatively frequent in our Chilean cohort (allele frequency: 15.3%; TT genotype frequency: 2.3%) and showed a positive correlation with Native American (primarily Mapuche) ethnic ancestry (Figure 2). It is important to state that these frequencies correspond to the population of the city of Santiago (Chile) and may vary across northern and southern Chilean sub-regions due to known geographic clines in genetic ancestry [33,34]. Consequently, our findings from this urban pediatric cohort in Santiago cannot be strictly generalized to the entire Chilean population. In contrast, the 125Phe T-allele was virtually absent in non-American populations (Table 2) [35,46]. In a recent genome-wide study of plasma metformin concentrations after an acute metformin challenge, higher drug concentrations were observed in individuals who were older, had a lower estimated glomerular filtration rate, and had a lower body mass index [47]. Several African ancestry-specific genetic variants were also significantly associated with the outcome. However, the overrepresentation of European and African ancestry groups and the limited representation of admixed Americans may have reduced the statistical power to identify associations involving MATE1 [47]. Currently, MATE1 and other metformin-related transporters are not included in the pharmacogenetic panel to prevent adverse drug reactions [48]. Despite the rarity of MALA, it is important to emphasize that the confirmed functional effect of p.Leu125Phe homozygotes (2.3% in Chile), the relatively high frequency in our population, its link with Amerindian ancestry, and the high burden of type 2 diabetes in our country are valid arguments for evaluating future possible pharmacovigilance of this genetic variant in high-risk groups in Chile.

In summary, previous research indicates that the SLC47A1 (MATE1) p.Leu125Phe (L125F; C>T) variant impairs MATE1-mediated metformin transport, as supported by *in vivo* studies (in one human and in mice) [35,37], *in vitro* studies (in HEK-293 transfected cells) [36], and *in silico* analyses [35]. This decreased-function effect may lead to differential renal elimination of metformin, systemic drug accumulation, and increased lactatemia, potentially representing a risk factor for MALA. The main finding of the present study is a relatively high frequency of the decreased-function variant 125Phe in the SLC47A1 gene (MATE1) among Chileans (15.3%, including 2.3% homozygotes), as previously reported in Mexico [35]. In contrast, this variant was virtually absent in non-American populations. The population-specific enrichment of SLC47A1 rs77474263 (p.Leu125Phe) underscores its potential relevance to metformin pharmacogenetics in Chileans and possibly in other admixed Latin American populations.

## Figures and Tables

**Table 1:** Genes related to metformin pharmacokinetics.

Protein	Gene	Location/Effect	Physiological Process
OCT1	*SLC22A1*	Basolateral membrane of enterocytes, hepatocytes, and renal epithelium	Intestinal absorption (major role), hepatic transport and renal secretion (minor roles)
OCT2	*SLC22A2*	Basolateral membrane of renal epithelial cells, allowing the metformin elimination process.	Renal elimination
OCT3	*SLC22A3*	Apical membrane of the enterocyte (absorption from the intestinal lumen) and in the hepatocyte.	Intestinal absorption (major role) and hepatic transport (minor role)
PMAT	*SLC29A4*	Apical membrane of the enterocyte, allowing absorption from the intestinal lumen.	Intestinal absorption
MATE1	*SLC47A1*	Apical membrane of renal cells, allowing renal elimination	Renal elimination
MATE2-K	*SLC47A2*	Apical membrane of renal cells, allowing renal elimination.	Renal elimination

**Table 2:** Allele frequency of functional missense gene variants related to metformin pharmacokinetics in Chile and worldwide.

Gene	Amino Acid Change	rs ID	Minor Allele Frequency (MAF) (%)							
			Chile	African	Admixed American	Native American	European	Central/ South Asian	East Asian	Polyphen Bioinformatic Prediction (Score)
*SLC22A1*	p.R61C	rs12208357	3.2	0	2.6	0	5.3	3.9	0.5	Probably damaging (0.99)
	p.G401S	rs34130495	1.1	0	0.9	0	3.0	0	0	Probably damaging (1.0)
	p.G465R	rs34059508	0.8	0	0.8	0.8	0	1.4	0	Probably damaging (0.98)
*SLC47A1*	p.L125F	rs77474263	15.3	0	11.5	13.0	0	0	0.7	Probably damaging (1.0)

Legend to table: No gene variants in SLC22A2/SLC22A3/SLC29A4/SLC47A2 fulfilled the selection criteria. Chilean data are estimated from the present study (GOCS). Allele frequencies were obtained from the Human Genome Diversity Project (HGDP) (accessed 26 August, 2026), as reported in the Genome Aggregation Database (gnomAD; https://gnomad.broadinstitute.org/; accessed on 1 September 2026). PolyPhen-2 score ranges: 0.0 to 0.15: Predicted to be benign or neutral; 0.15 to 0.85: Predicted to be possibly damaging; 0.85 to 1.0: Predicted to be probably damaging. No homozygotes were found for rs12208357 (860 R61 homozygotes; 58 heterozygotes), rs34130495 (897 G40 homozygotes; 21 heterozygotes), and rs34059508 (903 G465 homozygotes; 15 heterozygotes). The distribution of rs77474263 (p.L125F; C>T) genotypes is shown in Figure 2 (CC, n = 659; CT, n = 238; TT, n = 21). All genetic polymorphisms agreed with the Hardy–Weinberg equilibrium in the Chilean population (*p* = 0.32 for rs12208357, *p* = 0.73 for rs34130495; *p* = 0.82 for rs34059508, and *p* = 0.93 for rs77474263).

## Data Availability

All the information associated with this work can be found throughout the text. Additional details should be requested from the authors.

## Supplementary Materials

The following supporting information can be downloaded at: https://www.mdpi.com/article/10.3390/genes17091114/s1, Figure S1: Flow chart of GOCS participants and genotyping quality control (QC); Table S1: Average proportion of global ancestry among GOCS participants.
