# Supplementary material for: Genetic Variants Involved in Metformin Pharmacokinetics in the Chilean Population
Source: Genes (Basel). 2026 Sep 14;17(9):1114. doi: 10.3390/genes17091114 (PMC13606663; doi:10.3390/genes17091114)
Supplement: Supplementary file 1 [file genes-17-01114-s001.zip › genes-4487320-supplementary.pdf]

SUPPLEMENTARY MATERIAL

Genetic Variants Involved in Metformin Pharmacokinetics in the Chilean Population

José P. Miranda 1,2, Gigliola L. Alberti 3, Ana Pereira 4, Juan Cristóbal Gana 3, and José L. Santos 1,5,6

1. Department of Nutrition, Diabetes and Metabolism. School of Medicine. Pontificia Universidad Católica de Chile. Santiago, Chile.

2. Dirección de Desarrollo Médico y Académico, Bupa Chile, Santiago, Chile

3. Department of Pediatric Gastroenterology and Nutrition, Division of Pediatrics. School of Medicine, Pontificia Universidad Católica de Chile. Santiago, Chile.

4. Institute of Nutrition and Food Technology (INTA). University of Chile

5. Department of Health Sciences, Institute for Sustainability and Food Chain Innovation (IS-FOOD), Public University of Navarre, Pamplona, Spain.

6. Navarra Institute for Health Research (IdiSNA), Pamplona, Spain

7 . School of Medicine, Pontificia Universidad Católica de Chile, Santiago, Chile

Correspondence: to: José L. Santos, Department of Health Sciences, Institute for Sustainability and Food Chain Innovation (IS-FOOD), Public University of Navarre, Pamplona, Spain. E-mail: joseluis.santos@unavarra.es

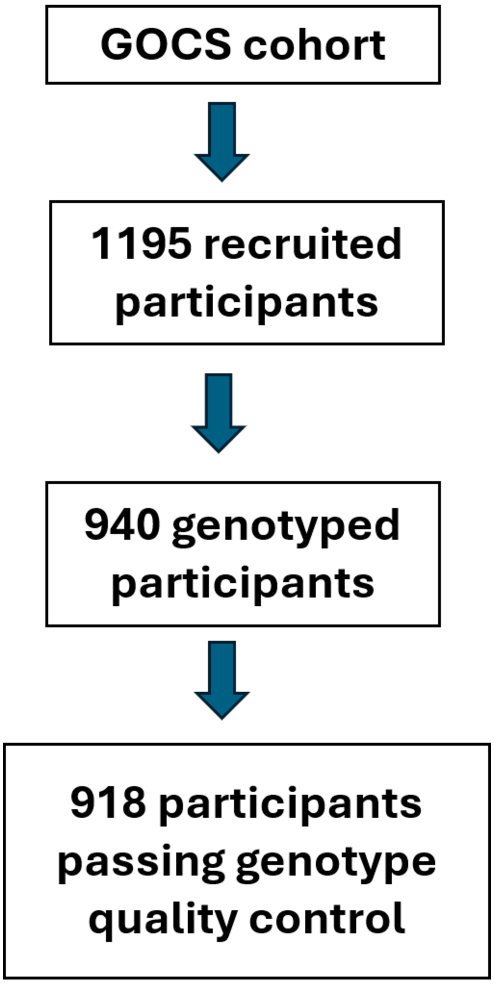

Figure S1. Flow chart of GOCS participants and genotyping quality control (QC). We performed genome-wide genotyping on 940 GOCS cohort participants available at the time of the study, and 8 duplicate genotyping controls from the Human Genome Diversity Cell Line Panel (HGDP-CEPH) using the Infinium MEGA Multhi-Ethnic Global BeadChip (Illumina. Inc). We loaded genotyping data into GenomeStudio v2.0.3 (Illumina. Inc) and called automatic clustering. From the total of samples analyzed, 14 with a call rate <0.98 were removed. The average genotyping concordance between the HGDP-CEPH Panel duplicate controls was 99.99% ± 0.02, and the total genotyping rate was 0.9993. The project was exported to PLINK format for further filtering. Using PLINK v1.9, we removed three more samples based on gender mismatch and three due to relatedness (IBD/IBS>0.2). Excluded variants had minor allele frequency (MAF) < 0.01, missing genotype data >5%, duplicated physical positions (one variant was kept from each duplicate pair), and deviations from the Hardy-Weinberg Equilibrium (HWE) (p<1 × 10−5). To rule out strand inconsistency, 58,236 transversions (A-T or C-G) were removed. After these filters, we obtained 918 participants (51% female) and a dataset of 700,344 autosomal variants in the GRCh37 human reference sequence.

Table S1. Average proportion of global ancestry among GOCS participants

Region	Population	Estimated Ancestry Percentage
Americas	Probably Mapuche#	55.3
	Peruvian	3.8
	Colombian, Surui, Maya, Karitiana, Pima, Mexican, Puerto Ricans	2.2
	**Total American**	61.4
Europe	Iberian, Basque, Sardinian, Toscani, French, Italian	25.0
	Finnish, British, Russian, Orcadian, Northern, and Western Europe	6.2
	Bedouin, Druze, Mozabite, Palestinian	4.8
	**Total European**	36.0
Africa	Pygmy, San, Bantu, Mende	0.05
	Esan, Yoruba, African Caribbean	0.17
	Gambian Mandinka	0.16
	Luhya	0.17
	**Total African**	0.55
East Asia	Kinh, Lahu, Cambodian, She, Miao, Tujia, Dai, Han	0.14
	Japanese, Hezhen, Daur, Oroqen, Yakut, Xibo, Mongola, Yi, Tu, Naxi, Uygur	0.20
	**Total East Asian**	0.34
Central and South Asia	Telegu, Tamil, Punjabi, Bengali, Gujarati, Burusho, Pathan, Sindhi	0.24
	Kalash, Brahui, Balochi, Makrani, Hazara	1.37
	**Total Central Asian**	1.60
Oceania	Melanesian, Papuan	0.13
	**Total Oceanian**	0.13

Legend to table S1: Proportions are presented at the Region, and Population-level estimated with K=15 using ADMIXTURE#. See below the section “Global ancestry assessment among GOCS participants”

Global ancestry assessment among GOCS participants

As a result of the global ancestry analysis, we found that Native American (NAT) ancestry was separated into three subpopulations. One subpopulation grouped samples of Central American origin; a second subpopulation grouped the 1KGP participants of Peruvian origin (PEL), which we called the Andean subpopulation; and a third subpopulation, which we believe corresponds to Mapuche origin. We make this statement because most GOCS participants cluster in this group, and we previously determined that the main component of NAT origin in our cohort is Mapuche ethnicity, with a marginal contribution from Aymara ethnicity [33]. Considering this, constructing a reference panel for local ancestry based solely on genotypes from the 1KGP and HGDP projects would greatly bias our results, as the Mapuche ancestry component is practically non-existent among those participants. Therefore, we decided to include 16 GOCS participants with a higher proportion of NAT ancestry in our panel to account for Mapuche-origin haplotypes [34]. In the article of Miranda et al. (2024), it is stated that the GOCS cohort showed a global Native American (NAT) ethnic component that reached 61.4%. This regional component was separated into three populations, with the main contribution coming from the Native-American Mapuche population (55.3%) and low contributions from Andean (3.84%) and Central American (2.2%) origins. In the present paper, we represent the Native American (Mapuche) ancestry component in the same terms. See below Table S1 showing the average proportion of global ancestry among GOCS participants.
